# Higher ER load is not associated with better outcome in stage 1–3 breast cancer: a descriptive overview of quantitative HR analysis in operable breast cancer

**DOI:** 10.1007/s10549-019-05233-9

**Published:** 2019-04-17

**Authors:** I. Noordhoek, A. F. de Groot, D. Cohen, G. J. Liefers, J. E. A. Portielje, J. R. Kroep

**Affiliations:** 1 Department of Surgery, Leiden University Medical Centre, Albinusdreef 2, 2333 ZA Leiden, The Netherlands; 2 Department of Medical Oncology, Leiden University Medical Centre, Leiden, The Netherlands; 3 Department of Pathology, Leiden University Medical Centre, Leiden, The Netherlands

**Keywords:** Hormone receptor, Immunohistochemistry, Quantitative assessment, Endocrine therapy, Estrogen receptor, Progesterone receptor

## Abstract

**Purpose:**

In breast cancer, hormone receptor (HR) status is generally a qualitative measure; positive or negative. Quantitatively measured oestrogen and progesterone receptors (ER and PR) are frequently proposed prognostic and predictive markers, some guidelines even provide different treatment options for patients with strong versus weak expression.

**Aim:**

To evaluate quantitative HR load assessed by immunohistochemistry as a prognostic and predictive measure in stage 1–3 breast cancer.

**Methods:**

We reviewed all the available literature on quantitatively measured HRs using immunohistochemistry.

**Results:**

All included studies (*n* = 19) comprised a cohort of 30,754 patients. Only 2 out of 17 studies found a clear correlation between higher quantitative ER and better disease outcome. Only one trial examined quantitative ER both as prognostic and predictive marker and found no association between ER% and survival. Ten studies examined quantitative PR load, only two of those found a significant correlation between higher PR load and better disease outcome. Two trials examined quantitative PR both as prognostic and predictive marker, neither found any association between PR% and disease outcome.

**Conclusions:**

There is no clear evidence for using quantitatively assessed ER and PR as prognostic nor predictive marker in patients with stage 1–3 breast cancer. We recommend only using a qualitative HR status in future guidelines and treatment considerations.

**Electronic supplementary material:**

The online version of this article (10.1007/s10549-019-05233-9) contains supplementary material, which is available to authorized users.

## Introduction

Breast cancer is the most common type of cancer amongst women worldwide and the leading cause of cancer-specific death for women in Europe [1]. The oestrogen receptor (ER) and progesterone receptor (PR) expression are the oldest biomarkers in breast cancer [2, 3].

Different methods exist for determining the expression of hormone receptors (HRs). The tissue can be analysed using enzyme immunoassays (EIA), in which the amount of HRs is expressed in fmol/mg, defining HR positive as 15 fmol/mg or more [4, 5]. More recently, however, immunohistochemistry (IHC) has been the preferred method of staining hormone receptors. The number of cells expressing HRs is counted, generating a percentage of positive cells [6]. Different cut-off levels are used to determine whether a tumour is considered HR positive. Usually, a tumour is considered HR positive when more than 10% of the tumour cells express HRs [7, 8].

Furthermore, nuclei can be grouped into categories of negative, weak, moderate and strong nuclear staining to generate a continuous histoscore ranging from 0 to 300, calculated by multiplying the sum of the percentage of weakly stained cells times 1, moderately stained cells times 2, and strongly stained cells times 3 [9]. Tumours with a histoscore of 50 or more are usually considered HR positive.

Additionally, the Allred scoring system has been used, which is a semi-quantitative measure that takes into consideration the proportion of positive cells (scored on a scale of 0–5) and staining intensity (scored on a scale of 0–3). The sum of these produces a score between 0 and 8, and tumours with a score of 3 or more are usually considered HR positive [10].

Another semi-quantitative measure is the ER immunoreactive score (IRS), which also relies on the proportion and intensity. This produces a score between 0 and 12, considering tumours with a score of 2 or higher HR positive [11].

Already more than 15 years ago, IHC was proposed as the reference method by different boards and peer committees [12] and a dichotomous, qualitative scale of HR expression (i.e. “positive” or “negative”) was unanimously adopted [13]. This method remains the gold standard for HR expression evaluation [14].

Although it is generally claimed that tumours with strong ER and/or PR expression are more sensitive to endocrine therapy (ET), there is no clear definition of weak or strong ER/PR expression. The most recent guidelines as proposed by the St. Gallen International Expert Consensus Conference on the Primary Therapy of Early Breast Cancer in 2017, briefly mention high ER expression as a characteristic of a low risk tumour and vice versa, but fail to provide any definition or cut-off value to determine which tumours are in fact high in ER expression [14]. As there is no consensus on the value of quantitative HR expression analysis, it is not (yet) common practice to report on HR load in the clinical setting.

This systematic review gives an overview of the methods to quantitatively assess HR load, the predictive and prognostic value of determining the HR load, gives recommendations for clinical practice and discusses future developments for HR analysis and endocrine treatment.

## Methods

### Data searches and study selection

To obtain all the relevant literature, the electronic databases PubMed, Embase and Web of Science were searched in March 2018, using the keywords presented in Table 1. The complete search string for all databases can be found in supplementary Table 1. This search was updated in August 2018 and in January 2019. According to PRISMA guidelines for systematic reviews, two of the authors (IN and AFG) individually and independently screened the articles for predefined inclusion criteria [15]. These were stated as follows:

- The article was published in English in a peer reviewed journal;
- The article was a primary report of original data;
- The study concerned women diagnosed with stage 1–3 adenocarcinoma of the breast;
- The tumour’s ER and/or PR expression was analysed using IHC (the international gold standard);
- ER and/or PR expression was reported quantitatively (continuous) or semi-quantitatively (minimum of three groups);
- Within the subset of HR-positive cases, the (semi-)quantitative measure of ER and/or PR was analysed in association to the primary clinical endpoint.

**Table 1:** Key words used for data search

Category	Key words
Hormone receptors (1)	Estrogen receptor, progesterone receptor, oestrogen receptor, estradiol receptor, hormone receptor, ER, PR, HR
Quantitative measurement (2)	Quantitative, level of ER expression, level of PR expression
Endocrine treatment (3)	Endocrine treatment, hormone treatment, hormonal treatment, endocrine therapy, hormone therapy, hormonal therapy, tamoxifen, aromatase inhibitor, anastrozole, letrozole, exemestane
Breast cancer (4)	Breast neoplasms, breast cancer, breast carcinoma, breast tumour, breast malignancy, mammary cancer, mammary carcinoma
Combined search string	(1) AND (2) AND (3) AND (4) NOT (“Animals”[mesh] NOT “Humans”[mesh])
Results	March 2nd 2018
PubMed	431 results
Embase	470 results, of which 132 unique
Web of science	290 results, of which 87 unique
Results	August 2nd 2018
PubMed	440 results, of which nine new and unique
Embase	491 results, of which six new and unique
Web of science	295 results, of which one new and unique
Results	January 15th 2019
PubMed	452 results, of which 12 new and unique
Embase	523 results, of which 29 new and unique
Web of science	317 results, of which nine new and unique
Google Scholar	69 results, of which 61 new and unique
Results	Overall
	777 unique results

Only studies that the reviewers reached a consensus on were included. If needed, a third reviewer was consulted.

Due to the retrospective nature of most included studies, it was elected not to perform a risk of bias assessment. Each study was awarded a level of evidence according to the Oxford Centre of Evidence-Based Medicine [16].

### Data extraction

All data from the included studies were analysed and data regarding the following items were extracted:

- Number of participating patients;
- Method of HR expression determination;
- Method of HR expression quantification;
- Type of systemic treatment (ET and chemotherapy) and timing (adjuvant or neoadjuvant);
- Primary clinical endpoint and follow-up time;
- Association primary clinical endpoint to quantified HR expression.

Due to the heterogeneity of the included studies, data was not pooled, and no meta-analyses were performed.

## Results

### Characteristics of included studies

**Fig. 1:**
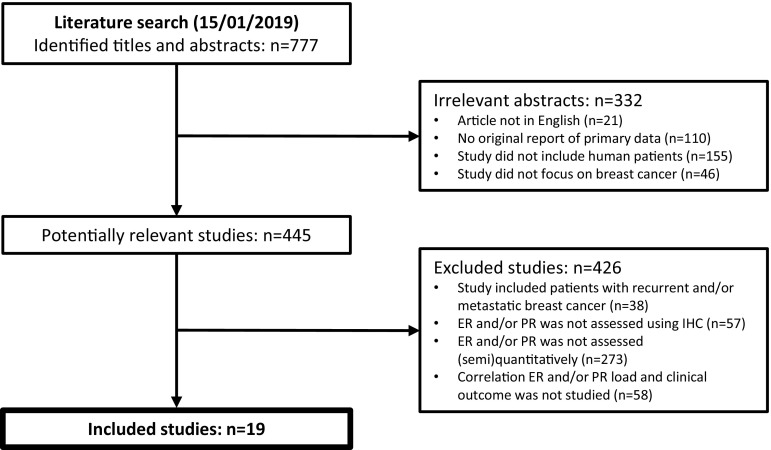
CONSORT diagram to account for excluded studies. *ER* Oestrogen receptor, *PR* Progesterone receptor, *IHC* Immunohistochemistry

Using the key words presented in Table 1, 777 unique articles were identified. After matching these to the inclusion criteria, 19 articles were included. The most common ground to exclude studies was not reporting ER and/or PR expression (semi-)quantitatively (*n* = 273) (Fig. 1). Combined, all included studies comprised a cohort of 30,754 patients.

### Quantitative assessment of HR expression

Of the nineteen included studies, six studies performed HR staining on whole-section slides of the tumour tissue, whereas nine studies first created TMAs, where several cores are taken off the tissue blocks. HR staining is then performed on these cores instead of on whole-section slides. In four studies, it was not specified how the staining was performed.

In five studies, a continuous quantitative measure (percentage or histoscore) was used to determine HR load, in four studies, patients were divided in groups of negative, low and high expression and in nine studies patients were divided in four or more groups according to the HR expression. In one study, both a continuous and a semi-quantitative measure was used.

### Systemic treatment of included patients

**Fig. 2:**
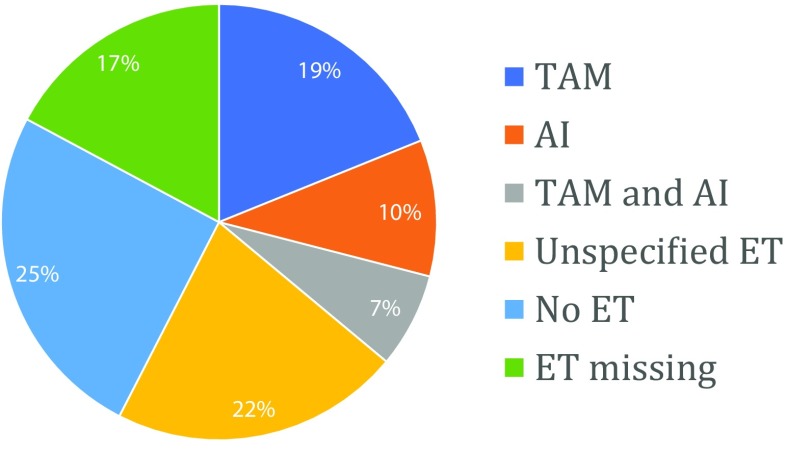
Distribution of types of endocrine therapy over patients. *TAM* Tamoxifen, *AI* Aromatase inhibitor, *ET* Endocrine therapy

Of all included patients, 5812 were treated with tamoxifen, 3111 were treated with an aromatase inhibitor (AI), 2164 were treated with a combination of tamoxifen and an AI, 6614 were treated with unspecified ET and 7769 patients did not receive any ET. For 5284 patients, it was not specified whether they received ET or not (Fig. 2). Additionally, 10,036 patients were treated with chemotherapy. For 7788 patients, it was not specified whether they received chemotherapy or not, 12,930 patients did not receive chemotherapy. Treatment with anti-HER2 medication was explicitly stated for only three patients [17]. Supplementary Table 2 provides detailed information on all included studies.

**Table 2:** Overview of results of the included articles studying oestrogen receptor (ER) load in 26,259 patients

References	Level of evidence and design	Number of patients	Outcome measure	Median FU (years)	Significant association
Bartlett 2011 [18]	2b, RCT	4325	DFS	5	Yes
Esslimani-Sahla 2004 [23]	3b, case–control	50	Recurrence	5	Yes
Dowsett 2008 [22]	1b, RCT	1856	DFS	5.7	Marginally
Ma 2013 [26]	3b, case–control	1206	BCSS	10	Marginally
Ryu 2018 [30]	3b, cohort	4948	OS	4.8	Marginally
Campbell 2016 [19]	2b, cohort	503	DFS	5.7	No
Chae 2011 [20]	2c, cohort	171	DFS	4.3	No
Chapman 2013 [21]	1b, RCT	345	DFS	9.7	No
Harigopal 2010 [24]	2b, RCT	1715	DFS	7.2	No
Hill 2017 [25]	3b, case–control	1098	OS	7.8	No
Mazouni 2010 [27]	1b, cohort	797	OS	6.3	No
Morgan 2011 [28]	3b, cohort	563	OS	10	No
Prabhu 2014 [17]	2b, cohort	231	DFS	2.4	No
Prat 2013 [29]	4, cohort	701	DRFS	12.5	No
Regierer 2011 [11]	2b, cohort	3971	RFS	5	No
Turbin 2008 [31]	2b, cohort	3484	BCSS	12.5	No
Zhang 2014 [32]	3b, cohort	295	OS	5	No

In case of statistically significant associations, a higher ER load is associated with better clinical outcome. Level of evidence, according to the Oxford Centre of Evidence-Based Medicine [18]

*FU* Follow-up, *DFS* Disease-free survival, *BCSS* Breast cancer-specific survival, *OS* Overall survival, *DRFS* Distant recurrence-free survival, *RFS* Recurrence-free survival

### Overall association ER load and clinical outcome

In 17 of the 19 included studies, the ER load was analysed, in a total of 26,259 patients with stage 1–3 breast cancer patients (Table 2) [11, 17–32]. In 11 studies, HR-negative patients were also included, all reported associations between the ER load and the primary outcome measure regard the subset of HR-positive cases only. Disease-free survival (DFS) was used as primary outcome measure in seven studies, overall survival (OS) was used in five studies, recurrence in three studies and breast cancer-specific mortality in two studies.

When studying ER load as a continuous measure (either using percentage or histoscore, *n* = 6), a higher ER load was found statistically significantly associated with better clinical outcome in two studies, marginally significantly associated in one study, and three studies did not find a significant association between higher ER load and better clinical outcome.

When dividing patients in three groups, i.e. ER-negative, low ER and high ER expression (*n* = 4), three studies did not find a significantly longer DFS for patients in the high ER expression group than patients in the low ER expression group, one study only found a marginally significant association between longer OS and higher ER expression.

When dividing patients in four or more groups based on ER expression (*n* = 8), seven studies did not find a significantly better clinical outcome for patients with a higher ER expression and one study only found a marginally significant association between better clinical outcome and higher ER expression.

These results are summarised in Table 2.

### Using ER load as a prognostic and/or predictive marker

There was only one randomised trial that compared patients with and without ET, which could be used to analyse the prognostic and predictive properties of the quantitative ER load without the risk of bias due to treatment indication. This study by Chapman et al. (*n* = 345) [21] used a continuous quantitative measure, stained on TMAs and found no significant correlation between higher ER percentages and longer DFS in the overall study population (*p* = 0.24). They did not find any association between higher ER load and longer DFS in the subgroup that was randomised to receive no adjuvant ET and thus conclude that the quantitative ER load cannot be used as a prognostic marker. They also did not find any association between higher ER load and longer DFS in the subgroup of patients that was treated with adjuvant ET and, therefore, conclude that quantitative ER load is not an adequate predictive marker for sensitivity to ET, either.

### Overall association PR load and clinical outcome

Of the 19 included studies, ten studies analysed PR load in 14,161 early breast cancer patients (Table 3) [18–24, 29, 33, 34]. In six studies, HR-negative patients were also included, all reported associations between the PR load and the primary outcome measure regard the subset of HR-positive cases only. DFS was used as primary outcome measure in six studies, three studies used recurrence as primary outcome and one study used breast cancer-specific mortality.

**Table 3:** Overview of results of the included articles studying progesterone receptor (PR) load in 14,161 patients

References	Level of evidence and design	Number of patients	Outcome measure	Median FU (years)	Significant association
Bartlett 2011 [18]	2b, RCT	4325	DFS	5	Yes
Dowsett 2008 [22]	1b, RCT	1856	DFS	5.7	Yes
Prat 2013 [29]	4, cohort	701	DRFS	12.5	Marginally
Campbell 2016 [19]	2b, cohort	503	DFS	5.7	No
Chae 2011 [20]	2c, cohort	171	DFS	4.3	No
Chapman 2013 [21]	1b, RCT	345	DFS	9.7	No
Esslimani-Sahla 2004 [23]	3b, case–control	50	Recurrence	5	No
Harigopal 2010 [9]	2b, RCT	1715	DFS	7.2	No
Liu 2010 [33]	2b, cohort	4046	BCSS	10	No
Nordenskjold 2016 [34]	2b, RCT	449	Recurrence	18	No

In case of statistically significant associations, a higher PR load is associated with better clinical outcome. Level of evidence, according to the Oxford Centre of Evidence-Based Medicine [16]

*FU* Follow-up, *DFS* Disease-free survival, *DRFS* Distant recurrence-free survival, *BCSS* Breast cancer-specific survival

When studying PR load as a continuous measure (*n* = 6), a higher PR load was found to be significantly associated with better clinical outcome in two studies, a higher PR load was found marginally significantly associated with better clinical outcome in one study, and three studies did not find any association between PR load and clinical outcome.

When dividing patients in PR-negative, low PR and high PR expression groups (*n* = 2), DFS was not significantly longer in the high PR expression group than in the low PR expression group.

When dividing patients in four or more groups based on their PR expression (*n* = 3), clinical outcome was not significantly better for a higher PR load.

These results are summarised in Table 3.

### Using PR load as a prognostic and/or predictive marker

There were two randomised trials comparing patients with and without ET, which could be used to analyse the prognostic and predictive properties of the quantitative PR load without risk of bias [21, 34]. Both studies randomised patients between tamoxifen or no adjuvant ET and used TMAs to stain the PR.

The study by Chapman et al. (*n* = 345) [21] used a continuous quantitative measure and found no association between continuous higher PR percentage and longer DFS in the overall randomised study population (*p* = 0.04; uncorrected for multiple testing). They did not find any association between higher PR load and longer DFS in the subgroup that received no adjuvant ET. They also did not find any association between higher PR load and longer DFS in the subgroup of patients that was treated with adjuvant ET.

The study by Nordenskjold et al. (*n* = 449) [34] divided patients in seven groups based on the number of positive PR staining cells and did not find an association between the PR percentage groups and the occurrence of disease recurrences in the overall study population. They found no association between higher PR load and less disease recurrences within the subgroup of patients that did and did not receive ET, either.

Thus, both studies concluded that quantitative PR load is not an adequate tool to determine the prognosis of early breast cancer patients, nor to predict sensitivity to ET.

### Interaction between ER and PR

Of the eight studies that examined both the ER and PR load, only two studied the interaction between ER and PR load. The study by Campbell et al. [19] found a statistically significant interaction between the quantitative ER and PR load, and only found a significant association between higher PR load and better outcome in those patients that also had a higher ER load. The study by Harigopal et al. [24] found a moderate interaction between continuous quantitative ER and PR percentage (Pearson *r *= 0.43, *p* < 0.0001).

This suggests that the quantitative PR load is not independently associated with outcome, but only in relation to the quantitative ER load.

## Discussion

Many efforts have been made to identify biomarkers or profiles in breast cancer patients capable of predicting sensitivity to endocrine treatment and the risk of recurrence after treatment is discontinued [35, 36]. One of these methods is the quantitative assessment of ER and PR expression, i.e. the ER and PR load, instead of merely assigning tumours an ER and PR-positive or negative status [37].

This review concludes that in patients with an ER-positive tumour (defined as ER > 10%), a higher ER load as assessed by IHC is not correlated to better outcome, and no evidence could be found for using quantitative ER load as a prognostic marker. In other words, patients with a higher ER load (e.g. 100%) do not inherently have a better prognosis than patients with a lower ER load (e.g. 20%). Furthermore, no evidence could be found for using quantitative ER load as a predictive marker, i.e. patients with a higher ER load do not have more benefit of ET than patients with a lower ER load.

This review also concludes that in patients with an HR-positive tumour, higher PR load does not seem to be correlated to better outcome. Based on the included studies, quantitative PR load is not a suitable prognostic marker; patients with a higher PR load do not inherently have a better prognosis than patients with a lower PR load, nor is it suitable as a predictive marker. Furthermore, PR load seems to be interacted with ER load and is therefore not recognised as an independent predictor.

One of the included studies, by Esslimani-Sahla [23], found an unusually high number of recurrences and only found an association between recurrence and ER load when examining ERβ, not when examining ERα. As this is the only study that specifically examines ERβ, it is somewhat of an outlier, and its results should be interpreted with caution.

In this analysis, only studies that examined the HR expression using IHC were included. This method is the gold standard for determining HR status and other methods, such as EIA or mRNA expression profiles are not routinely used in clinical practice. Specifically, we have not focussed on articles studying EIA to determine HR status, as this method is outdated and is not routinely used in the current clinical practice. This also ensures that the included studies create a homogenous cohort. Even still, different methods were used to stain the HRs, such as staining on whole-section slides or on TMAs, though this did not seem to influence the outcome; studies staining on TMAs were not less likely to find a correlation between HR load and outcome than studies staining on whole-section slides.

Studies also differed in their way of quantitatively measuring HR load; some studies used a continuous percentage or histoscore, some studies used groups of HR-negative, low HR expression and high HR expression and some divided patients in four or more groups based on Allred score or percentage. This does have an influence on the outcome. Studies were more likely to find a positive association between HR load and outcome if a continuous score was used. However, using a continuous quantitative measure to assess HR expression is questioned by several articles. Interobserver variability is high, and samples get assigned different HR percentages depending on the pathologist and the lab it was reviewed in [38]. Most importantly, staining breast cancer tissue using IHC does not allow for precise enough measurement of HR load to generate a continuous score and can only quantify into negative, weak positive and strong positive [7, 39, 40]. The problem with this approach is defining “weak” and “strong”. A lack of generally accepted definition results in pathologists and papers choosing their own definition, making it difficult to compare multiple studies. Furthermore, and as-mentioned previously, the St. Gallen Consensus makes a distinction between high and low ER expression but fails to provide any definition or cut-off value to determine which tumours are in fact high in ER expression [14]. The St. Gallen Consensus does not mention high and low PR expression at all.

Based on the results of this review, we propose using both ER and PR expression only as a qualitative measure; defining tumours with < 1–10% of cells expressing this receptor as negative, and tumours with more than 1–10% of cells expressing the receptor as positive [17, 41]. Using a continuous quantitative measure does not seem feasible without centralised, unambiguous and clear pathological measurement. The implications for the daily clinical practise of pathologists are that more detailed information on the HR status beyond “positive” or “negative” should no longer be provided, to prevent oncologists subconsciously or instinctively making different treatment decisions based on this information. Since there is no evidence for different treatment strategies, providing extra information is both unnecessary and undesirable.

Simultaneously, one can speculate whether there is any added value of measuring the PR status at all. It is generally accepted that there is no such thing as an ER-negative/PR-positive tumour [42, 43]. Since the quantitative PR load is correlated to the quantitative ER load and PR load is inversely correlated to the histological grade of the tumour [19, 24], the question arises whether PR status provides any additional prognostic information, when ER status, grade and potentially a proliferation factor such as ki-67 is known. Likewise, when examining guidelines on adjuvant treatment, they do not propose different treatment strategies for tumours that are ER positive/PR positive compared to tumours that are ER positive/PR negative [8, 14]. Therefore, if the PR status is unlikely to change the course of treatment, it could be considered wasteful and excessive to continue measuring it [44, 45]. It might be worthwhile to focus future research on the independent contribution of PR status using multivariable models.

Gene expression profiling can be used to identify two inherently different entities within breast cancer, known as luminal-A and luminal-B. These are intrinsic molecular subtypes that reflect a different tumour biology and disease prognosis. Unfortunately, neither subtype is predictive for a better response to ET [29, 46–50]. Moreover, the gene expression profiles used to differentiate between these subtypes are expensive and not universally available and the added value for daily clinical practice, in particular for which subgroup of patients, is still debated [35]. For these reasons, researchers have tried to approach the distinction between these molecular subtypes using IHC, which resulted in subtypes called luminal-A-like and luminal-B-like [29]. When defining IHC-based luminal-A-like tumours as HR positive, HER2 negative and ki-67 below 14%, approximately 81–85% of luminal-A tumours were correctly identified as luminal-A-like. However, approximately 35–52% of luminal-B tumours were incorrectly identified as luminal-A-like. When expanding the definition of luminal-A-like to HR positive, HER2 negative, ki-67 below 14% and PR above 20%, the specificity improves somewhat but not enough to accurately discriminate between the two subtypes [29, 48, 50].

With these considerations, and the lack of prognostic and predictive value of IHC assessed quantitative ER and PR load as shown in this review, the distinction between IHC-based luminal-A-like and luminal-B-like tumours should not be used to tailor treatment decisions for women with HR-positive stage 1–3 breast cancer.

### Future perspectives

All in all, identifying the early breast cancer patients that could benefit most from ET remains a challenge, as more than half of all patients with an ER-positive breast cancer will not respond to ET [51]. Considering the frequent and often severe side effects of ET, an improved upfront selection of likely responders may lower the treatment burden. Since quantitative measurement of HR does not seem an appropriate instrument for identifying these patients, the oncologic community is searching for much needed other means to predict response to ET. One potential method to identify patients is to measure the activity of the ER pathway to distinguish in which patients the oestrogen receptor is not only expressed but also active and thus a suitable target for ET [52]. The use of predictive biomarkers in the neoadjuvant ET setting will be clearer after results of ongoing trials become available. Potentially, response to neoadjuvant therapy can be measured at a per patient level using postoperative pathology, bypassing the need for predictive markers altogether.

## Conclusion

There is no clear evidence for using quantitative ER and PR load assessed by immunohistochemistry as a prognostic measure nor as a predictive marker for response to ET in patients with stage 1–3 breast cancer. Immunohistochemistry is the gold standard for measuring HR status but should only be used to distinguish HR-negative and HR-positive tumours. Gene expression profiles have prognostic value for women with ER-positive disease, early response evaluation to neoadjuvant therapy holds promise in the prediction of long-term response to endocrine therapy.

## Electronic supplementary material

Below is the link to the electronic supplementary material.

Supplementary material 1 (DOCX 56 kb)

## References

[1] Allemani C, Matsuda T, Di Carlo V, Harewood R, Matz M, Nikšić M, Bonaventure A, Valkov M, Johnson CJ, Estève J, Ogunbiyi OJ (2018). Global surveillance of trends in cancer survival 2000-14 (CONCORD-3): analysis of individual records for 37,513,025 patients diagnosed with one of 18 cancers from 322 population-based registries in 71 countries. Lancet.

[2] Dekker TJ, Smit VT, Hooijer GK, Van de Vijver MJ, Mesker WE, Tollenaar RA, Nortier JW, Kroep JR (2013). Reliability of core needle biopsy for determining ER and HER2 status in breast cancer. Ann Oncol.

[3] Dekker TJA, ter Borg S, Hooijer GKJ, Meijer SL, Wesseling J, Boers JE, Schuuring E, Bart J, van Gorp J, Bult P, Riemersma SA, van Deurzen CHM, Sleddens HFBM, Mesker WE, Kroep JR, Smit VTHBM, van de Vijver MJ (2015). Quality assessment of estrogen receptor and progesterone receptor testing in breast cancer using a tissue microarray-based approach. Breast Cancer Res Treat.

[4] Heuson JC, Longeval E, Mattheiem WH, Deboel MC, Sylvester RJ, Leclercq G (1977). Significance of quantitative assessment of estrogen receptors for endocrine therapy in advanced breast cancer. Cancer.

[5] McGuire WL (1980). Steroid hormone receptors in breast cancer treatment strategy. Recent Prog Horm Res.

[6] Khoshnoud MR, Lofdahl B, Fohlin H, Fornander T, Stal O, Skoog L, Bergh J, Nordenskjold B (2011). Immunohistochemistry compared to cytosol assays for determination of estrogen receptor and prediction of the long-term effect of adjuvant tamoxifen. Breast Cancer Res Treat.

[7] Hammond ME, Hayes DF, Wolff AC, Mangu PB, Temin S (2010). American society of clinical oncology/college of american pathologists guideline recommendations for immunohistochemical testing of estrogen and progesterone receptors in breast cancer. J Oncol Pract.

[8] NBCA (2017) Richtlijn mammacarcinoom: BEPALINGEN VAN HORMOONRECEPTOREN EN HER2. Oncoline

[9] Andersen J, Orntoft TF, Poulsen HS (1988). Immunohistochemical demonstration of estrogen receptors (ER) in formalin-fixed, paraffin-embedded human breast cancer tissue by use of a monoclonal antibody to ER. J Histochem Cytochem.

[10] Qureshi A, Pervez S (2010). Allred scoring for ER reporting and it’s impact in clearly distinguishing ER negative from ER positive breast cancers. JPMA.

[11] Regierer AC, Wolters R, Kurzeder C, Wockel A, Novopashenny I, Possinger K, Wischnewsky MB, Kreienberg R (2011). High estrogen receptor expression in early breast cancer: chemotherapy needed to improve RFS?. Breast Cancer Res Treat.

[12] Bast RC, Ravdin P, Hayes DF, Bates S, Fritsche H, Jessup JM, Kemeny N, Locker GY, Mennel RG, Somerfield MR (2001). 2000 update of recommendations for the use of tumor markers in breast and colorectal cancer: clinical practice guidelines of the American Society of Clinical Oncology. J Clin Oncol.

[13] Goldhirsch A, Wood WC, Gelber RD, Coates AS, Thurlimann B, Senn HJ (2003). Meeting highlights: updated international expert consensus on the primary therapy of early breast cancer. J Clin Oncol.

[14] Curigliano G, Burstein HJ, Winer EP, Gnant M, Dubsky P, Loibl S, Colleoni M, Regan MM, Piccart-Gebhart M, Senn HJ, Thürlimann B, André F, Baselga J, Bergh J, Bonnefoi H, Brucker SY, Cardoso F, Carey L, Ciruelos E, Cuzick J, Denkert C, Di Leo A, Ejlertsen B, Francis P, Galimberti V, Garber J, Gulluoglu B, Goodwin P, Harbeck N, Hayes DF, Huang CS, Huober J, Khaled H, Jassem J, Jiang Z, Karlsson P, Morrow M, Orecchia R, Osborne KC, Pagani O, Partridge AH, Pritchard K, Ro J, Rutgers EJT, Sedlmayer F, Semiglazov V, Shao Z, Smith I, Toi M, Tutt A, Viale G, Watanabe T, Whelan TJ, Xu B (2017). De-escalating and escalating treatments for early-stage breast cancer: the St. Gallen International Expert Consensus Conference on the Primary Therapy of Early Breast Cancer 2017. Ann Oncol.

[15] Moher D, Liberati A, Tetzlaff J, Altman DG (2009). Preferred reporting items for systematic reviews and meta-analyses: the PRISMA statement. BMJ.

[16] Medicine CfE-B (2009) Oxford centre for evidence-based medicine—levels of evidence (March 2009)

[17] Prabhu JS, Korlimarla A, Desai K, Alexander A, Raghavan R, Anupama CE, Dendukuri N, Manjunath S, Correa M, Raman N, Kalamdani A, Prasad MSN, Gopinath KS, Srinath BS, Sridhar TS (2014). A majority of low (1-10%) er positive breast cancers behave like hormone receptor negative tumors. J Cancer.

[18] Bartlett JM, Brookes CL, Robson T, van de Velde CJ, Billingham LJ, Campbell FM, Grant M, Hasenburg A, Hille ET, Kay C, Kieback DG, Putter H, Markopoulos C, Kranenbarg EM, Mallon EA, Dirix L, Seynaeve C, Rea D (2011). Estrogen receptor and progesterone receptor as predictive biomarkers of response to endocrine therapy: a prospectively powered pathology study in the Tamoxifen and Exemestane Adjuvant Multinational trial. J Clin Oncol.

[19] Campbell EJ, Tesson M, Doogan F, Mohammed ZMA, Mallon E, Edwards J (2016). The combined endocrine receptor in breast cancer, a novel approach to traditional hormone receptor interpretation and a better discriminator of outcome than ER and PR alone. Br J Cancer.

[20] Chae BJ, Bae JS, Yim HW, Lee A, Song BJ, Jeon HM, Chun MH, Jung SS (2011). Measurement of ER and PR status in breast cancer using the QuantiGene2.0 assay. Pathology.

[21] Chapman JA, Nielsen TO, Ellis MJ, Bernard P, Chia S, Gelmon KA, Pritchard KI, Maitre A, Goss PE, Leung S, Shepherd LE, Bramwell VH (2013). Effect of continuous statistically standardized measures of estrogen and progesterone receptors on disease-free survival in NCIC CTG MA1.2 trial and BC cohort. Breast Cancer Res.

[22] Dowsett M, Allred C, Knox J, Quinn E, Salter J, Wale C, Cuzick J, Houghton J, Williams N, Mallon E, Bishop H, Ellis I, Larsimont D, Sasano H, Carder P, Cussac AL, Knox F, Speirs V, Forbes J, Buzdar A (2008). Relationship between quantitative estrogen and progesterone receptor expression and human epidermal growth factor receptor 2 (HER-2) status with recurrence in the Arimidex, Tamoxifen, Alone or in Combination trial. J Clin Oncol.

[23] Esslimani-Sahla M, Simony-Lafontaine J, Kramar A, Lavaill R, Mollevi C, Warner M, Gustafsson JA, Rochefort H (2004). Estrogen receptor beta (ER beta) level but not its ER beta cx variant helps to predict tamoxifen resistance in breast cancer. Clin Cancer Res.

[24] Harigopal M, Barlow WE, Tedeschi G, Porter PL, Yeh IT, Haskell C, Livingston R, Hortobagyi GN, Sledge G, Shapiro C, Ingle JN, Rimm DL, Hayes DF (2010). Multiplexed assessment of the Southwest Oncology Group-directed Intergroup Breast Cancer Trial S9313 by AQUA shows that both high and low levels of HER2 are associated with poor outcome. Am J Pathol.

[25] Hill DA, Barry M, Wiggins C, Nibbe A, Royce M, Prossnitz E, Lomo L (2017). Estrogen receptor quantitative measures and breast cancer survival. Breast Cancer Res Treat.

[26] Ma HY, Lu YN, Marchbanks PA, Folger SG, Strom BL, McDonald JA, Simon MS, Weiss LK, Malone KE, Burkman RT, Sullivan-Halley J, Deapen DM, Press MF, Bernstein L (2013). Quantitative measures of estrogen receptor expression in relation to breast cancer-specific mortality risk among white women and black women. Breast Cancer Res.

[27] Mazouni C, Bonnier P, Goubar A, Romain S, Martin PM (2010). Is quantitative oestrogen receptor expression useful in the evaluation of the clinical prognosis? Analysis of a homogeneous series of 797 patients with prospective determination of the ER status using simultaneous EIA and IHC. Eur J Cancer.

[28] Morgan DAL, Refalo NA, Cheung KL (2011). Strength of ER-positivity in relation to survival in ER-positive breast cancer treated by adjuvant tamoxifen as sole systemic therapy. Breast.

[29] Prat A, Cheang MC, Martin M, Parker JS, Carrasco E, Caballero R, Tyldesley S, Gelmon K, Bernard PS, Nielsen TO, Perou CM (2013). Prognostic significance of progesterone receptor-positive tumor cells within immunohistochemically defined luminal A breast cancer. J Clin Oncol.

[30] Ryu JM, Choi HJ, Kim I, Lee SK, Yu J, Kim J-E, Kang B-I, Lee JE, Nam SJ, Kim SW (2018). Only estrogen receptor “positive” is not enough to predict the prognosis of breast cancer. Breast Cancer Res Treat.

[31] Turbin DA, Leung S, Cheang MC, Kennecke HA, Montgomery KD, McKinney S, Treaba DO, Boyd N, Goldstein LC, Badve S, Gown AM, van de Rijn M, Nielsen TO, Gilks CB, Huntsman DG (2008). Automated quantitative analysis of estrogen receptor expression in breast carcinoma does not differ from expert pathologist scoring: a tissue microarray study of 3,484 cases. Breast Cancer Res Treat.

[32] Zhang Z, Wang J, Skinner KA, Shayne M, Hajdu SI, Bu H, Hicks DG, Tang P (2014). Pathological features and clinical outcomes of breast cancer according to levels of oestrogen receptor expression. Histopathology.

[33] Liu S, Chia SK, Mehl E, Leung S, Rajput A (2010). Progesterone receptor is a significant factor associated with clinical outcomes and effect of adjuvant tamoxifen therapy in breast cancer patients. Breast Cancer Res Treat.

[34] Nordenskjold A, Fohlin H, Fornander T, Lofdahl B, Skoog L, Stal O (2016). Progesterone receptor positivity is a predictor of long-term benefit from adjuvant tamoxifen treatment of estrogen receptor positive breast cancer. Breast Cancer Res Treat.

[35] Blok EJ, Bastiaannet E, van den Hout WB, Liefers GJ, Smit V, Kroep JR, van de Velde CJH (2018). Systematic review of the clinical and economic value of gene expression profiles for invasive early breast cancer available in Europe. Cancer Treat Rev.

[36] Viale G, Ghioni M, Mastropasqua MG (2010). Traditional molecular markers and response to adjuvant endocrine or trastuzumab-based therapies. Curr Opin Oncol.

[37] van de Water W, Fontein DB, van de Nes JG, Bartlett JM, Hille ET, Putter H, Robson T, Liefers GJ, Roumen RM, Seynaeve C, Dirix LY, Paridaens R, Kranenbarg EM, Nortier JW, van de Velde CJ (2013). Influence of semi-quantitative oestrogen receptor expression on adjuvant endocrine therapy efficacy in ductal and lobular breast cancer—a TEAM study analysis. Eur J Cancer.

[38] Reisenbichler ES, Lester SC, Richardson AL, Dillon DA, Ly A, Brock JE (2013). Interobserver concordance in implementing the 2010 ASCO/CAP recommendations for reporting er in breast carcinomas A demonstration of the difficulties of consistently reporting low levels of ER expression by manual quantification. Am J Clin Pathol.

[39] Nadji M (2008). Quantitative immunohistochemistry of estrogen receptor in breast cancer: “much ado about nothing!”. Appl Immunohistochem Mol Morphol.

[40] Schnitt SJ (2006). Estrogen receptor testing of breast cancer in current clinical practice: what’s the question?. J Clin Oncol.

[41] Deyarmin B, Kane JL, Valente AL, van Laar R, Gallagher C, Shriver CD, Ellsworth RE (2013). Effect of ASCO/CAP guidelines for determining ER status on molecular subtype. Ann Surg Oncol.

[42] Foley NM, Coll JM, Lowery AJ, Hynes SO, Kerin MJ, Sheehan M, Brodie C, Sweeney KJ (2018). Re-appraisal of estrogen receptor negative/progesterone receptor positive (ER-/PR+) breast cancer phenotype: true subtype or technical artefact?. Pathol Oncol Res.

[43] Kunc M, Biernat W, Senkus-Konefka E (2018). Estrogen receptor-negative progesterone receptor-positive breast cancer—”Nobody’s land” or just an artifact?. Cancer Treat Rev.

[44] EBCTCG (1998). Tamoxifen for early breast cancer: an overview of the randomised trials. Early Breast Cancer Trialists’ Collaborative Group. Lancet (London).

[45] Olivotto IA, Truong PT, Speers CH, Bernstein V, Allan SJ, Kelly SJ, Lesperance ML (2004). Time to stop progesterone receptor testing in breast cancer management. J Clin Oncol.

[46] Ellis MJ, Suman VJ, Hoog J, Lin L, Snider J, Prat A, Parker JS, Luo J, DeSchryver K, Allred DC, Esserman LJ, Unzeitig GW, Margenthaler J, Babiera GV, Marcom PK, Guenther JM, Watson MA, Leitch M, Hunt K, Olson JA (2011). Randomized phase II neoadjuvant comparison between letrozole, anastrozole, and exemestane for postmenopausal women with estrogen receptor-rich stage 2 to 3 breast cancer: clinical and biomarker outcomes and predictive value of the baseline PAM50-based intrinsic subtype–ACOSOG Z1031. J Clin Oncol.

[47] Nielsen TO, Parker JS, Leung S, Voduc D, Ebbert M, Vickery T, Davies SR, Snider J, Stijleman IJ, Reed J, Cheang MC, Mardis ER, Perou CM, Bernard PS, Ellis MJ (2010). A comparison of PAM50 intrinsic subtyping with immunohistochemistry and clinical prognostic factors in tamoxifen-treated estrogen receptor-positive breast cancer. Clin Cancer Res.

[48] Parker JS, Mullins M, Cheang MC, Leung S, Voduc D, Vickery T, Davies S, Fauron C, He X, Hu Z, Quackenbush JF, Stijleman IJ, Palazzo J, Marron JS, Nobel AB, Mardis E, Nielsen TO, Ellis MJ, Perou CM, Bernard PS (2009). Supervised risk predictor of breast cancer based on intrinsic subtypes. J Clin.

[49] Prat A, Ellis MJ, Perou CM (2011). Practical implications of gene-expression-based assays for breast oncologists. Nat Rev Clin Oncol.

[50] Viale G, Snoo FA, Slaets L, Bogaerts J, Veer L, Rutgers EJ, Piccart-Gebhart MJ, Stork-Sloots L, Glas A, Russo L, Dell’Orto P, Tryfonidis K, Litiere S, Cardoso F (2018). Immunohistochemical versus molecular (BluePrint and MammaPrint) subtyping of breast carcinoma. Breast Cancer Res Treat.

[51] EBCTCG (2015). Aromatase inhibitors versus tamoxifen in early breast cancer: patient-level meta-analysis of the randomised trials. Lancet (London, England).

[52] Verhaegh W, van Ooijen H, Inda MA, Hatzis P, Versteeg R, Smid M, Martens J, Foekens J, van de Wiel P, Clevers H, van de Stolpe A (2014). Selection of personalized patient therapy through the use of knowledge-based computational models that identify tumor-driving signal transduction pathways. Can Res.

