# Supplementary material for: Higher ER load is not associated with better outcome in stage 1–3 breast cancer: a descriptive overview of quantitative HR analysis in operable breast cancer
Source: Breast Cancer Res Treat. 2019 Apr 17;176(1):27–36. doi: 10.1007/s10549-019-05233-9 (PMC6548750; doi:10.1007/s10549-019-05233-9)
Supplement: Supplementary file 1 — Supplementary material 1 (DOCX 56 kb) [file 10549_2019_5233_MOESM1_ESM.docx]

| ***Database*** | ***Search string*** |
| --- | --- |
| *PubMed* | (("Receptors, Estrogen"[Mesh] OR "Receptors, Progesterone"[Mesh] OR "estrogen receptor"[tiab] OR "oestrogen receptor"[tiab] OR "progesterone receptor"[tiab] OR "estrogen receptors"[tiab] OR "oestrogen receptors"[tiab] OR "progesterone receptors"[tiab] OR "estradiol receptor"[tiab] OR "estradiol receptor"[tiab] OR "ER"[tiab] OR "PR"[tiab] OR "HR"[tiab] OR (("hormone receptor"[tiab] OR "hormone receptors"[tiab]) AND (estrogen*[tiab] OR oestrogen*[tiab] OR progesteron*[tiab]))) **AND** ("Quantitative"[tiab] OR quantitativ*[tiab]) **AND** ("Endocrine treatment"[tiab] OR "hormone treatment"[tiab] OR "hormonal treatment"[tiab] OR "Endocrine therapy"[tiab] OR "hormone therapy"[tiab] OR "hormonal therapy"[tiab] OR "Hormones/therapeutic use"[Mesh] OR "Hormones/administration and dosage"[Mesh] OR "Hormones/drug effects"[Mesh] OR "tamoxifen"[tiab] OR "Tamoxifen"[Mesh] OR "aromatase inhibitor"[tiab] OR "aromatase inhibitors"[tiab] OR "Aromatase Inhibitors"[Mesh] OR "anastrozole"[tiab] OR "letrozole"[tiab] OR "exemestane"[tiab]) **AND** ("Breast Neoplasms"[Mesh] OR "breast cancer"[tiab] OR "breast cancers"[tiab] OR "breast carcinoma"[tiab] OR "breast carcinomas"[tiab] OR "breast tumor"[tiab] OR "breast tumors"[tiab] OR "breast tumour"[tiab] OR "breast tumours"[tiab] OR "breast neoplasm"[tiab] OR "breast neoplasms"[tiab] OR "breast malignancy"[tiab] OR "breast malignancies"[tiab] OR "mammary cancer"[tiab] OR "mammary cancers"[tiab] OR "mammary carcinoma"[tiab] OR "mammary carcinomas"[tiab] OR "mammary tumor"[tiab] OR "mammary tumors"[tiab] OR "mammary tumour"[tiab] OR "mammary tumours"[tiab] OR "mammary neoplasm"[tiab] OR "mammary neoplasms"[tiab] OR "mammary malignancy"[tiab] OR "mammary malignancies"[tiab]) **NOT** ("Animals"[mesh] NOT "Humans"[mesh])) |
| *Embase* | ((exp "Estrogen receptor"/ OR "Progesterone Receptor"/ OR "estrogen receptor".ti,ab OR "oestrogen receptor".ti,ab OR "progesterone receptor".ti,ab OR "estrogen receptors".ti,ab OR "oestrogen receptors".ti,ab OR "progesterone receptors".ti,ab OR "estradiol receptor".ti,ab OR "estradiol receptor".ti,ab OR "ER".ti,ab OR "PR".ti,ab OR "HR".ti,ab OR (("hormone receptor".ti,ab OR "hormone receptors".ti,ab) AND (estrogen*.ti,ab OR oestrogen*.ti,ab OR progesteron*.ti,ab))) **AND** ("Quantitative".ti,ab OR quantitativ*.ti,ab OR "Level of ER expression".af OR "Levels of ER expression".af OR "Level of Estrogen receptor expression".af OR "Levels of Estrogen receptor expression".af OR "Level of Oestrogen receptor expression".af OR "Levels of Oestrogen receptor expression".af OR "Level of PR expression".af OR "Levels of PR expression".af OR "Level of Progesterone receptor expression".af OR "Levels of Progesterone receptor expression".af) **AND** ("Endocrine treatment".ti,ab OR "hormone treatment".ti,ab OR "hormonal treatment".ti,ab OR "Endocrine therapy".ti,ab OR "hormone therapy".ti,ab OR "hormonal therapy".ti,ab OR exp "hormonal therapy"/ OR "tamoxifen".ti,ab OR "Tamoxifen"/ OR "aromatase inhibitor".ti,ab OR "aromatase inhibitors".ti,ab OR exp "Aromatase Inhibitor"/ OR "anastrozole".ti,ab OR "letrozole".ti,ab OR "exemestane".ti,ab) **AND** (exp "Breast Cancer"/ OR "breast cancer".ti,ab OR "breast cancers".ti,ab OR "breast carcinoma".ti,ab OR "breast carcinomas".ti,ab OR "breast tumor".ti,ab OR "breast tumors".ti,ab OR "breast tumour".ti,ab OR "breast tumours".ti,ab OR "breast neoplasm".ti,ab OR "breast neoplasms".ti,ab OR "breast malignancy".ti,ab OR "breast malignancies".ti,ab OR "mammary cancer".ti,ab OR "mammary cancers".ti,ab OR "mammary carcinoma".ti,ab OR "mammary carcinomas".ti,ab OR "mammary tumor".ti,ab OR "mammary tumors".ti,ab OR "mammary tumour".ti,ab OR "mammary tumours".ti,ab OR "mammary neoplasm".ti,ab OR "mammary neoplasms".ti,ab OR "mammary malignancy".ti,ab OR "mammary malignancies".ti,ab) **AND** exp "Humans"/) |
| *Web of Science* | (ti=("Estrogen receptor" OR "Progesterone Receptor" OR "estrogen receptor" OR "oestrogen receptor" OR "progesterone receptor" OR "estrogen receptors" OR "oestrogen receptors" OR "progesterone receptors" OR "estradiol receptor" OR "estradiol receptor" OR "ER" OR "PR" OR "HR" OR (("hormone receptor" OR "hormone receptors") AND (estrogen* OR oestrogen* OR progesteron*))) **AND** ts=("Quantitative" OR quantitativ* OR "Level of ER expression" OR "Levels of ER expression" OR "Level of Estrogen receptor expression" OR "Levels of Estrogen receptor expression" OR "Level of Oestrogen receptor expression" OR "Levels of Oestrogen receptor expression" OR "Level of PR expression" OR "Levels of PR expression" OR "Level of Progesterone receptor expression" OR "Levels of Progesterone receptor expression") **AND** ts=("Endocrine treatment" OR "hormone treatment" OR "hormonal treatment" OR "Endocrine therapy" OR "hormone therapy" OR "hormonal therapy" OR "hormonal therapy" OR "tamoxifen" OR "Tamoxifen" OR "aromatase inhibitor" OR "aromatase inhibitors" OR "Aromatase Inhibitor" OR "anastrozole" OR "letrozole" OR "exemestane") **AND** ts=("Breast Cancer" OR "breast cancer" OR "breast cancers" OR "breast carcinoma" OR "breast carcinomas" OR "breast tumor" OR "breast tumors" OR "breast tumour" OR "breast tumours" OR "breast neoplasm" OR "breast neoplasms" OR "breast malignancy" OR "breast malignancies" OR "mammary cancer" OR "mammary cancers" OR "mammary carcinoma" OR "mammary carcinomas" OR "mammary tumor" OR "mammary tumors" OR "mammary tumour" OR "mammary tumours" OR "mammary neoplasm" OR "mammary neoplasms" OR "mammary malignancy" OR "mammary malignancies") **NOT** ti=(veterinary OR rabbit OR rabbits OR animal OR animals OR mouse OR mice OR rodent OR rodents OR rat OR rats OR pig OR pigs OR porcine OR horse* OR equine OR cow OR cows OR bovine OR goat OR goats OR sheep OR ovine OR canine OR dog OR dogs OR feline OR cat OR cats)) |
| ***Results*** | **March 2^nd^ 2018** |
| *PubMed* | 431 results |
| *Embase* | 470 results, of which 132 unique |
| *Web of science* | 290 results, of which 87 unique |
| ***Results*** | **August 2^nd^ 2018** |
| *PubMed* | 440 results, of which 9 new and unique |
| *Embase* | 491 results, of which 6 new and unique |
| *Web of science* | 295 results, of which 1 new and unique |
| ***Results*** | **January 15^th^ 2019** |
| *PubMed* | 452 results, of which 12 new and unique |
| *Embase* | 523 results, of which 29 new and unique |
| *Web of science* | 317 results, of which 9 new and unique |
| *Google Scholar* | 69 results, of which 61 new and unique |
| ***Results*** | **Overall** |
|  | 777 unique results |

Supplementary table 1: Search strings used for data search.

| **Reference** | **Level of evidence and design** | **N** | **Pathology methodology** | **Systemic treatment** | **Median FU (years)** | **Was ER load studied?** | **Was PR load studied?** |
| --- | --- | --- | --- | --- | --- | --- | --- |
| Bartlett, 2011 [1] | 2b, RCT | 4325 | Staining on TMA.  Continuous histoscore (1-300). | **ET 1:** TAM, then exemestane, n=2164.  **ET 2:** Exemestane alone, n=2161.  **Chemotherapy**: n=US. | 5 , DFS | Yes | Yes |
| Campbell, 2016 [2] | 2b, cohort | 503 | Staining on TMA.  Allred scoring system (negative, low or high). | **ET 1:** TAM, n=368. **ET 2:** No ET, n=135.  **Chemotherapy**: n=208. | 5.7, DFS | Yes | Yes |
| Chae, 2011 [3] | 2c, cohort | 171 | Staining on whole-section slides.  Allred scoring system (negative, low or high). | **ET 1**: TAM alone, n=US. **ET 2**: AI alone, n=US. **ET 3**: TAM + GnRH-analogue, n=US.  **ET 4**: TAM, then AI, n=US.  **Chemotherapy**: n=114. | 4.3, DFS | Yes | Yes |
| Chapman, 2013 [4] | 1b, RCT | 345 | Staining on TMA.  Continuous visual score (0-100%). | **ET 1:** TAM, n=US. **ET 2:** No ET, n=US.  **Chemotherapy:** n=US. | 9.7, DFS | Yes | Yes |
| Dowsett, 2008 [5] | 1b, RCT | 1856 | Staining on TMA.  Continuous histoscore (1-300). | **ET 1:** TAM, n=906. **ET 2:** Anastrozole, n=950. **Chemotherapy:** n=167. | 5.7, DFS | Yes | Yes |
| Esslimani-Sahla, 2004 [6] | 3b, case-control | 50 | Staining on whole-section slides.  Continuous visual score (1-100%). | **ET:** TAM, n=50.  **Chemotherapy**: n=0. | 5, recurrence | Yes | Yes |
| Harigopal, 2010 [7] | 2b, RCT | 1715 | Staining on TMA.  Continuous visual score (0-100%) and quartiles. | **ET 1**: TAM, n=US. **ET 2**: No ET, n=US.  **Chemotherapy:** n=1,715. | 7.2, DFS | Yes | Yes |
| Hill, 2017 [8] | 3b, case-control | 1098 | Staining on TMA.  Visual score groups (1-59%, 60-89%, 90%, 91-96%, ≥97%). | **ET**: n=US.  **Chemotherapy**: n=US. | 7.8, OS | Yes | No |
| Liu, 2010 [9] | 2b, cohort | 4046 | Staining on TMA.  Visual score groups (<1%, 1-25%, 26-75%, ≥76%). | **ET 1**: TAM, n=1,606. **ET 2**: Other ET, n=12. **ET 3**: No ET, n=2,428.  **Chemotherapy**: n=1,045. | 10, BCSS | No | Yes |
| Ma, 2013 [10] | 3b, case-control | 1206 | Staining on whole-section slides.  Visual score groups (<1%, 1-39%, 40-59%, 60-79%, ≥80%). | **ET**: n=US.  **Chemotherapy**: n=US. | 10, BCSS | Yes | No |
| Mazouni, 2010 [11] | 1b, cohort | 797 | Staining method US.  Visual score groups (negative, weak, moderate, high). | **ET**: n=US.  **Chemotherapy**: n=US. | 6.3, OS | Yes | No |
| Morgan, 2011 [12] | 3b, cohort | 563 | Staining on whole-section slides.  Histoscore groups (1-50, 51-100, 101-200, ≥201). | **ET**: TAM, n=563.  **Chemotherapy**: n=0. | 10, OS | Yes | No |
| Nordenskjold, 2016 [13] | 2b, RCT | 449 | Staining on TMA.  Visual score groups (<1%, 1-9%, 10-24%, 25-49%, 50-74%, 75-89%, ≥90%). | **ET 1**: TAM, n=233. **ET 2**: No ET, n=216.  **Chemotherapy**: n=0. | 18, recurrence | No | Yes |
| Prabhu, 2014 [14] | 2b, cohort | 231 | Staining on whole-section slides.  Visual score groups (<1%, 1-10%, ≥11%). | **ET 1:** ET, n=143. **ET 2:** No ET, n=88.  **Chemotherapy**: n=204. | 2.4, DFS | Yes | No |
| Prat, 2013 [15] | 4, cohort | 701 | Staining method US.  Continuous histoscore (1-300). | **ET**: TAM, n=701.  **Chemotherapy**: n=0. | 12.5, recurrence | Yes | Yes |
| Regierer, 2011 [16] | 2b, cohort | 3971 | Staining method US.  IRS groups (negative, weak, moderate, high). | **ET 1**: ET, n=2463. **ET 2:** No ET, n=1508.  **Chemotherapy**: n=1844. | 5, RFS | Yes | No |
| Ryu, 2018 [17] | 3b, cohort | 4948 | Staining method US.  Allred scoring system (negative, low or high). | **ET 1:** ET, n=2463. **ET 2:** No ET, n=1224. **ET 3:** ET missing, n=123.  **Chemotherapy:** n=3646. | 4.8, OS | Yes | No |
| Turbin, 2008 [18] | 2b, cohort | 3484 | Staining on TMA.  Visual score groups (<1%, 1-24%, 25-75%, ≥76%). | **ET 1:** TAM, n=1385. **ET 2:** No ET, n=2099. **Chemotherapy**: n=920. | 12.5, BCSS | Yes | No |
| Zhang, 2014 [19] | 3b, cohort | 295 | Staining on whole-section slides.  Visual score groups (<1%, 1-10%, 11-50%, 51-70%, ≥71%). | **ET 1**: ET, n=224. **ET 2:** No ET, n=71. **Chemotherapy:** n=173. | 5, OS | Yes | No |

Supplementary table 2: Overview of methods of the included articles, studying the hormone receptor load in 30,745 patients with stage 1-3 breast cancer .

Level of evidence, according to the Oxford Centre of Evidence Based Medicine [20]. N = Number of included patients. FU = Follow-up. ER = Oestrogen receptor. PR = Progesterone receptor. RCT = Randomised controlled trial. TMA = Tissue micro-array. HR = Hormone receptor. ET = Endocrine therapy. TAM = Tamoxifen. US = Unspecified. DFS = Disease free survival. AI = Aromatase inhibitor. GnRH = Gonadotropin-releasing hormone. OS = Overall survival. BCSS = Breast cancer specific survival. IRS = Immunoreactive score. RFS = Recurrence free survival.

References:

1. Bartlett JM, Brookes CL, Robson T, van de Velde CJ, Billingham LJ, Campbell FM, Grant M, Hasenburg A, Hille ET, Kay C, Kieback DG, Putter H, Markopoulos C, Kranenbarg EM, Mallon EA, Dirix L, Seynaeve C, Rea D (2011) Estrogen receptor and progesterone receptor as predictive biomarkers of response to endocrine therapy: a prospectively powered pathology study in the Tamoxifen and Exemestane Adjuvant Multinational trial. J Clin Oncol 29 (12):1531-1538. doi:10.1200/jco.2010.30.3677

2. Campbell EJ, Tesson M, Doogan F, Mohammed ZMA, Mallon E, Edwards J (2016) The combined endocrine receptor in breast cancer, a novel approach to traditional hormone receptor interpretation and a better discriminator of outcome than ER and PR alone. British Journal of Cancer 115 (8):967-973

3. Chae BJ, Bae JS, Yim HW, Lee A, Song BJ, Jeon HM, Chun MH, Jung SS (2011) Measurement of ER and PR status in breast cancer using the QuantiGene2.0 assay. Pathology 43 (3):248-253

4. Chapman JA, Nielsen TO, Ellis MJ, Bernard P, Chia S, Gelmon KA, Pritchard KI, Le Maitre A, Goss PE, Leung S, Shepherd LE, Bramwell VH (2013) Effect of continuous statistically standardized measures of estrogen and progesterone receptors on disease-free survival in NCIC CTG MA.12 Trial and BC Cohort. Breast Cancer Res 15 (4):R71. doi:10.1186/bcr3465

5. Dowsett M, Allred C, Knox J, Quinn E, Salter J, Wale C, Cuzick J, Houghton J, Williams N, Mallon E, Bishop H, Ellis I, Larsimont D, Sasano H, Carder P, Cussac AL, Knox F, Speirs V, Forbes J, Buzdar A (2008) Relationship between quantitative estrogen and progesterone receptor expression and human epidermal growth factor receptor 2 (HER-2) status with recurrence in the Arimidex, Tamoxifen, Alone or in Combination trial. J Clin Oncol 26 (7):1059-1065. doi:10.1200/jco.2007.12.9437

6. Esslimani-Sahla M, Simony-Lafontaine J, Kramar A, Lavaill R, Mollevi C, Warner M, Gustafsson JA, Rochefort H (2004) Estrogen receptor beta (ER beta) level but not its ER beta cx variant helps to predict tamoxifen resistance in breast cancer. Clin Cancer Res 10 (17):5769-5776. doi:10.1158/1078-0432.ccr-04-0389

7. Harigopal M, Barlow WE, Tedeschi G, Porter PL, Yeh IT, Haskell C, Livingston R, Hortobagyi GN, Sledge G, Shapiro C, Ingle JN, Rimm DL, Hayes DF (2010) Multiplexed assessment of the Southwest Oncology Group-directed Intergroup Breast Cancer Trial S9313 by AQUA shows that both high and low levels of HER2 are associated with poor outcome. The American journal of pathology 176 (4):1639-1647. doi:10.2353/ajpath.2010.090711

8. Hill DA, Barry M, Wiggins C, Nibbe A, Royce M, Prossnitz E, Lomo L (2017) Estrogen receptor quantitative measures and breast cancer survival. Breast Cancer Research and Treatment 166 (3):855-864

9. Liu S, Chia SK, Mehl E, Leung S, Rajput A, ... (2010) Progesterone receptor is a significant factor associated with clinical outcomes and effect of adjuvant tamoxifen therapy in breast cancer patients. Breast cancer research-áΓÇª

10. Ma HY, Lu YN, Marchbanks PA, Folger SG, Strom BL, McDonald JA, Simon MS, Weiss LK, Malone KE, Burkman RT, Sullivan-Halley J, Deapen DM, Press MF, Bernstein L (2013) Quantitative measures of estrogen receptor expression in relation to breast cancer-specific mortality risk among white women and black women. Breast Cancer Research 15 (5)

11. Mazouni C, Bonnier P, Goubar A, Romain S, Martin PM (2010) Is quantitative oestrogen receptor expression useful in the evaluation of the clinical prognosis? Analysis of a homogeneous series of 797 patients with prospective determination of the ER status using simultaneous EIA and IHC. Eur J Cancer 46 (15):2716-2725. doi:S0959-8049(10)00445-4 [pii];10.1016/j.ejca.2010.05.021 [doi]

12. Morgan DAL, Refalo NA, Cheung KL (2011) Strength of ER-positivity in relation to survival in ER-positive breast cancer treated by adjuvant tamoxifen as sole systemic therapy. The Breast

13. Nordenskjold A, Fohlin H, Fornander T, Lofdahl B, Skoog L, Stal O (2016) Progesterone receptor positivity is a predictor of long-term benefit from adjuvant tamoxifen treatment of estrogen receptor positive breast cancer. Breast Cancer Res Treat 160 (2):313-322. doi:10.1007/s10549-016-4007-5

14. Prabhu JS, Korlimarla A, Desai K, Alexander A, Raghavan R, Anupama CE, Dendukuri N, Manjunath S, Correa M, Raman N, Kalamdani A, Prasad MSN, Gopinath KS, Srinath BS, Sridhar TS (2014) A majority of low (1-10%) er positive breast cancers behave like hormone receptor negative tumors. Journal of Cancer 5 (2):156-165

15. Prat A, Cheang MC, Martin M, Parker JS, Carrasco E, Caballero R, Tyldesley S, Gelmon K, Bernard PS, Nielsen TO, Perou CM (2013) Prognostic significance of progesterone receptor-positive tumor cells within immunohistochemically defined luminal A breast cancer. J Clin Oncol 31 (2):203-209. doi:10.1200/jco.2012.43.4134

16. Regierer AC, Wolters R, Kurzeder C, Wockel A, Novopashenny I, Possinger K, Wischnewsky MB, Kreienberg R (2011) High estrogen receptor expression in early breast cancer: chemotherapy needed to improve RFS? Breast Cancer Research and Treatment 128 (1):273-281

17. Ryu JM, Choi HJ, Kim I, Lee SK, Yu J, Kim J-E, Kang B-I, Lee JE, Nam SJ, Kim SW (2018) Only estrogen receptor "positive" is not enough to predict the prognosis of breast cancer. Breast Cancer Research and Treatment 172 (3):627-636

18. Turbin DA, Leung S, Cheang MC, Kennecke HA, Montgomery KD, McKinney S, Treaba DO, Boyd N, Goldstein LC, Badve S, Gown AM, van de Rijn M, Nielsen TO, Gilks CB, Huntsman DG (2008) Automated quantitative analysis of estrogen receptor expression in breast carcinoma does not differ from expert pathologist scoring: a tissue microarray study of 3,484 cases. Breast Cancer Res Treat 110 (3):417-426. doi:10.1007/s10549-007-9736-z

19. Zhang Z, Wang J, Skinner KA, Shayne M, Hajdu SI, Bu H, Hicks DG, Tang P (2014) Pathological features and clinical outcomes of breast cancer according to levels of oestrogen receptor expression. Histopathology 65 (4):508-516. doi:10.1111/his.12412

20. Medicine CfE-B (2009) Oxford Centre for Evidence-based Medicine – Levels of Evidence (March 2009).
